# Conservation of the structure and function of bacterial tryptophan synthases

**DOI:** 10.1107/S2052252519005955

**Published:** 2019-05-29

**Authors:** Karolina Michalska, Jennifer Gale, Grazyna Joachimiak, Changsoo Chang, Catherine Hatzos-Skintges, Boguslaw Nocek, Stephen E. Johnston, Lance Bigelow, Besnik Bajrami, Robert P. Jedrzejczak, Samantha Wellington, Deborah T. Hung, Partha P. Nag, Stewart L. Fisher, Michael Endres, Andrzej Joachimiak

**Affiliations:** a Center for Structural Genomics of Infectious Diseases, University of Chicago, Chicago, IL 60367, USA; b Structural Biology Center, Biosciences Division, Argonne National Laboratory, Argonne, IL 60439, USA; c Midwest Center for Structural Genomics, Biosciences Division, Argonne National Laboratory, Argonne, IL 60439, USA; d Broad Institute of MIT and Harvard, Cambridge, MA 02141, USA; e Department of Biochemistry and Molecular Biology, University of Chicago, Chicago, IL 60367, USA

**Keywords:** allosteric regulation, crystal structure, enzyme inhibitors, tryptophan, catalysis, structure determination, protein structure, molecular recognition, X-ray crystallography, enzyme mechanisms, drug discovery, tryptophan synthase, *Streptococcus pneumoniae*, *Legionella pneumophila*, *Francisella tularensis*

## Abstract

Tryptophan biosynthesis is one of the most characterized processes in bacteria, in which the enzymes from *Salmonella typhimurium* and *Escherichia coli* serve as model systems. Tryptophan synthase (TrpAB) catalyzes the final two steps of tryptophan biosynthesis in plants, fungi and bacteria. This pyridoxal 5′-phosphate (PLP)-dependent enzyme consists of two protein chains, α (TrpA) and β (TrpB), functioning as a linear αββα heterotetrameric complex containing two TrpAB units. The reaction has a complicated, multistep mechanism resulting in the β-replacement of the hydroxyl group of l-serine with an indole moiety. Recent studies have shown that functional TrpAB is required for the survival of pathogenic bacteria in macrophages and for evading host defense. Therefore, TrpAB is a promising target for drug discovery, as its orthologs include enzymes from the important human pathogens *Streptococcus pneumoniae*, *Legionella pneumophila* and *Francisella tularensis*, the causative agents of pneumonia, legionnaires’ disease and tularemia, respectively. However, specific biochemical and structural properties of the TrpABs from these organisms have not been investigated. To fill the important phylogenetic gaps in the understanding of TrpABs and to uncover unique features of TrpAB orthologs to spearhead future drug-discovery efforts, the TrpABs from *L. pneumophila*, *F. tularensis* and *S. pneumoniae* have been characterized. In addition to kinetic properties and inhibitor-sensitivity data, structural information gathered using X-ray crystallo­graphy is presented. The enzymes show remarkable structural conservation, but at the same time display local differences in both their catalytic and allosteric sites that may be responsible for the observed differences in catalysis and inhibitor binding. This functional dissimilarity may be exploited in the design of species-specific enzyme inhibitors.

## Abstract 2

The tryptophan synthases from three human pathogens show remarkable structural conservation, but at the same time display local differences in both their catalytic and allosteric sites that may be responsible for the observed differences in catalysis and inhibitor binding. This functional dissimilarity may be exploited in the design of species-specific enzyme inhibitors.

## 1. Introduction   

**Figure 1:**
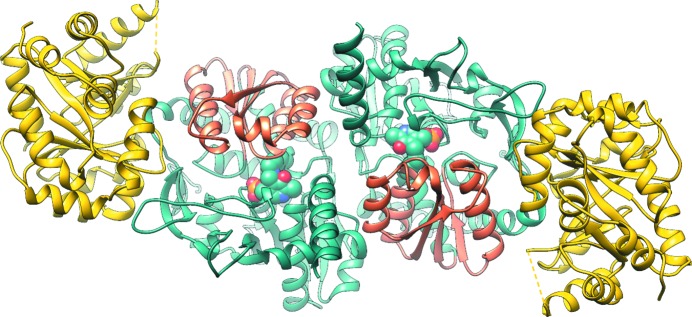
Overall structure of the tryptophan synthase αββα heterotetramer from *S. pneumoniae*. TrpA is shown in yellow and TrpB is shown in cyan, with the COMM domain shown in orange and the PLP cofactor depicted in a sphere representation.

**Figure 2:**
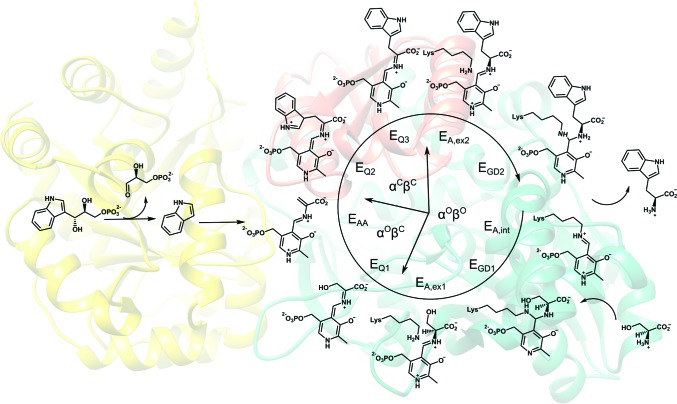
Enzymatic reaction of TrpAB. For TrpB, intermediate steps are shown (E_A,int_, internal aldimine; E_GD1_, geminal diamine; E_A,ex1_, external aldimine; E_Q1_, quinonoid; E_AA_, aminoacrylate; E_Q2_, quinonoid; E_A,ex2_, external aldimine; E_GD2_, geminal diamine).

Tryptophan synthase (TrpAB) is a pyridoxal 5′-phosphate (PLP)-dependent enzyme that participates in the final two steps of tryptophan synthesis in plants, fungi and bacteria (reviewed in Dunn, 2012 ▸; Raboni *et al.*, 2003 ▸, 2009 ▸; Dunn *et al.*, 2008 ▸). The enzyme consists of two protein chains, α (TrpA) and β (TrpB) (Crawford & Yanofsky, 1958 ▸), that operate as a linear αββα heterotetrameric complex containing two functional TrpAB units (Fig. 1 ▸). In bacteria, TrpA and TrpB are encoded by usually adjacent *trpA* and *trpB* genes that belong to the highly regulated tryptophan-biosynthesis operon (reviewed in Merino *et al.*, 2008 ▸). The TrpA subunit converts indole-3-glycerol phosphate (IGP) into glyceraldehyde 3-phosphate (G3P) and indole (IND) (Fig. 2 ▸). Subsequently, the latter product is utilized by TrpB, where it reacts with the l-serine (l-Ser) substrate to generate l-tryptophan (l-Trp). The reaction has a complicated, multistep mechanism involving enzyme–cofactor and substrate covalent adducts and results in the β-replacement of the hydroxyl group of l-Ser with the indole moiety (Fig. 2 ▸) (reviewed in Raboni *et al.*, 2009 ▸).

As originally shown for TrpAB from the Gram-negative *Salmonella typhimurium* (*St*TrpAB), TrpA adopts a canonical (β/α)_8_-barrel fold (also known as a TIM barrel) with numerous additional elements (Hyde *et al.*, 1988 ▸; Figs. 1 ▸ and 3 ▸). The active site is located at the top of the central β-barrel, with two acidic residues involved in catalysis: *St*Glu49 belonging to the αS2 strand and *St*Asp60 originating from loop αL2. Another structural element, loop αL6, serves as a lid closing over the binding pocket. TrpB represents a type II PLP-dependent enzyme with two domains, the N- and C-terminal domains, with the active site located in a cleft between them and carrying the covalently attached PLP cofactor. The N-terminal domain encompasses the so-called communication domain (COMM) that plays a key role in coordinating the activity of the two active sites (Schneider *et al.*, 1998 ▸). In the tetrameric arrangement, the TrpA and TrpB catalytic sites of the adjoining subunits are connected by a 25 Å long hydrophobic channel that facilities indole transport from TrpA to TrpB.

The TrpA- and TrpB-catalyzed chemical transformations are highly controlled by allosteric effects and other factors, for instance the binding of monovalent cations to TrpB, linked to substrate channeling. These molecular measures, together with other bacterial regulatory mechanisms (Merino *et al.*, 2008 ▸), are in place to ensure that the cellular resources are efficiently utilized to produce l-Trp, which is a scarce and most energetically expensive amino acid to biosynthesize (Akashi & Gojobori, 2002 ▸). The well documented ligand-induced reciprocal communication between subunits leading to the mutual activation involves conformational rearrangements. During the catalytic process, both TrpA and TrpB cycle between a low-activity open conformation (α^O^ or β^O^) and a high-activity closed state (α^C^ or β^C^) (Dunn, 2012 ▸), depending on the reaction state. The formation of the amino­acrylate Schiff-base intermediate, E_AA_, from l-Ser and PLP in TrpB triggers movement of the TrpB COMM domain towards a closed state (β^C^), which subsequently activates TrpA by closure of the αL6 loop (α^C^). In a reciprocal process, IGP substrate binding to TrpA promotes an α^C^ state, which in turn activates TrpB (β^C^). The two protein chains convert back to their open states when the l-Trp external aldimine, E_A,ex2_, is produced.

The availability of l-Trp, either supplied by the environment or synthesized *in cellulo*, is a prerequisite for bacterial survival. Some species rely heavily on external sources and maintain either no or only limited functionality of the l-Trp operon, while others preserve the complete system for *de novo* biosynthesis. The absence of the l-Trp biosynthetic pathway in animals and humans makes it a potentially attractive drug target for the treatment of bacterial diseases, even though the enzymes involved are only essential under certain conditions; that is, when exogenous l-Trp becomes depleted. Recent studies exploring these avenues showed that anthranilate syn­thase component I, TrpE (Zhang *et al.*, 2013 ▸), as well as functional tryptophan synthase are required for the survival of *Mycobacterium tuberculosis* in macrophage and mouse infection models, when an adaptive immune response triggers the expression of host indoleamine 2,3-dioxygenase (IDO-1), an enzyme responsible for l-Trp breakdown, or possibly even before this defense mechanism is mounted (Wellington *et al.*, 2017 ▸). Similar mechanisms inducing l-Trp starvation also function in lung-specific mouse infections with *Streptococcus pneumoniae* and *Francisella tularensis*, which are Gram-positive and Gram-negative bacteria, respectively. Under such conditions, the latter organism also requires TrpAB for growth (Peng & Monack, 2010 ▸). Other pathogens that utilize tryptophan biosynthesis to evade host defenses or even to highjack it for their own purposes include urogenital serovars of *Chlamydia trachomatis* (a Gram-negative obligate intracellular parasite), which employ a partly dysfunctional TrpAB to produce l-Trp from external sources of indole provided by coexisting bacteria (Caldwell *et al.*, 2003 ▸; Bonner *et al.*, 2014 ▸). The growing list of human pathogens in which the l-Trp biosynthetic pathway plays an important role extends beyond prokaryotes. For example, *Cryptosporidium* species (parasitic protozoa) inhabiting intestines encode bacteria-derived TrpB, which potentially acts in a similar fashion as it does in *C. trachomatis* (Sateriale & Striepen, 2016 ▸).

Specific biochemical and structural traits of the tryptophan synthases from these organisms have not been explored, with the recent exception of the *M. tuberculosis* ortholog. The structural and functional information gathered over the past 60 years has helped to explain the roles of individual residues in catalysis and allosteric regulation of the two active sites. Research has focused primarily on a prototypic tryptophan synthase from *S. typhimurium* (*St*TrpAB) and to a lesser extent those from *E. coli* (Heilmann, 1978 ▸; Lane & Kirschner, 1983 ▸; Drewe & Dunn, 1985 ▸, 1986 ▸; Houben & Dunn, 1990 ▸; Lim *et al.*, 1991 ▸) and *Pyrococcus furiosus* (Yamagata *et al.*, 2001 ▸; Ogasahara *et al.*, 2003 ▸; Hioki *et al.*, 2004 ▸; Lee *et al.*, 2005 ▸; Buller *et al.*, 2015 ▸; Heilmann, 1978 ▸; Lane & Kirschner, 1983 ▸; Drewe & Dunn, 1985 ▸, 1986 ▸; Houben & Dunn, 1990 ▸; Lim *et al.*, 1991 ▸). Tryptophan synthase has become a prototype system to study the peculiarities of allostery and substrate channeling (Hilario *et al.*, 2016 ▸; Ngo, Harris *et al.*, 2007 ▸; Ngo, Kimmich *et al.*, 2007 ▸; Niks *et al.*, 2013 ▸; Rhee *et al.*, 1996 ▸; Rowlett *et al.*, 1998 ▸; Spyrakis *et al.*, 2006 ▸). TrpA is also one of the model proteins that have been used to investigate protein-folding mechanisms (Wu & Matthews, 2002 ▸; Bilsel *et al.*, 1999 ▸; Yang *et al.*, 2007 ▸; Vadrevu *et al.*, 2008 ▸; Wu *et al.*, 2007 ▸; Michalska *et al.*, 2015 ▸). The sparsity of biochemical/structural investigations of other orthologs possibly stems from challenges in obtaining high-quality TrpAB samples and also from interest being focused on very detailed mechanistic aspects rather than on species-specific variations. Importantly, though, as shown by our recent study of *M. tuberculosis* TrpAB (*Mt*TrpAB; Wellington *et al.*, 2017 ▸), these so-far ignored differences, especially within the non­conserved tunnel lining, may have profound consequences for the discovery and design of new allosteric inhibitors.

Therefore, to fill the important phylogenetic gaps in our understanding of TrpABs and to uncover potential unique features of other orthologs to facilitate future drug-discovery efforts, we biochemically characterized three TrpABs from Gram-positive and Gram-negative pathogens: *Legionella pneumophila* Philadelphia, *F. tularensis* and *S. pneumoniae* (*LpPh*TrpAB, *Ft*TrpAB and *Sp*TrpAB, respectively). In addition to kinetic properties and inhibitor-binding capabilities, we also provide high-resolution structural information gathered using X-ray crystallography for the *Ft*TrpAB and *Sp*TrpAB complexes and for two α subunits: *LpPh*TrpA and that from *L. pneumophila* Paris (*LpPa*TrpA)*.*

## 2. Experimental procedures   

### 2.1. TrpAB gene cloning   

The gene cloning was performed as reported previously (Kim *et al.*, 2011 ▸). Briefly, *F. tularensis* Schu 4, *L. pneumophila* Philadelphia, *L. pneumophila* Paris and *S. pneumoniae* TIGR4 genomic DNAs were used as templates for PCR of the genes coding for the TrpA and TrpB subunits of tryptophan synthase. Vector-compatible primers for the amplification of the DNA fragments coding for the subunits were designed using an online tool (https://bioinformatics.anl.gov/targets/public_tools.aspx; Yoon *et al.*, 2002 ▸). The TrpA subunit peptides that were cloned were as follows: 1–269 for *Ft*TrpA, 1–272 for *LpPh*TrpA and *LpPa*TrpA, and 1–258 for *Sp*TrpA. The TrpB subunit peptides that were cloned were as follows: 1–396 for *Ft*TrpB, 13–396 for *LpPh*TrpB and 4–407 for *Sp*TrpB. Purified PCR products were treated with T4 DNA polymerase in the presence of dCTP (Eschenfeldt *et al.*, 2010 ▸) according to the vendor’s specification (New England Biolabs, Ipswich, Massachusetts, USA). The protruded DNA fragment for each of the TrpA subunits was mixed with T4 DNA polymerase-treated vector pMCSG68 (PSI:Biology-Materials Repository) to allow ligation-independent cloning (Aslanidis & Jong, 1990 ▸; Eschenfeldt *et al.*, 2009 ▸). Similarly, the protruded DNA fragment for each of the TrpB subunits was mixed with T4 DNA polymerase-treated vector pRSF with kanamycin resistance, which had an identical ligand-independent cloning site to pMCSG68. Both subunits from each genomic DNA were individually transformed into *E. coli* BL21-Gold (DE3) cells and grown in the presence of the corresponding antibiotic. A single colony of each transformant was picked, grown and induced with isopropyl β-d-1-thiogalactopyranoside (IPTG). The cell lysate was analyzed to confirm a protein of the correct molecular weight. The solubility of the TrpA subunit was analyzed via small-scale Ni^2+^-affinity purification and overnight TEV protease cleavage. Once the DNA sequences of the TrpA and TrpB subunits had been verified, both subunit plasmids from each genomic DNA were co-transformed into *E. coli* BL21-Gold (DE3) cells in LB medium containing ampicillin (150 µg ml^−1^) and kanamycin (25 µg ml^−1^). Co-transformed colonies were analyzed using Ni^2+^-affinity purification, and overnight TEV protease cleavage was performed to verify that the complex was soluble and stable.

### 2.2. Expression of TrpAB and purification for crystallization   

To express *Sp*TrpAB and *Ft*TrpAB, starter cultures were grown overnight at 37°C and 200 rev min^−1^ in LB medium with ampicillin (100 µg ml^−1^) and kanamycin (30 µg ml^−1^) supplemented with 40 m*M* K_2_HPO_4_. The following morning, LB–PO_4_–glucose (2 g per litre) medium with antibiotics was inoculated with the overnight cultures. After reaching an OD_600_ of 1.0 at 37°C, the *Sp*TrpAB cultures were transferred to 4°C and, after 1 h, to 18°C. After a subsequent 15 min incubation, the cultures were induced with 0.5 m*M* IPTG and incubated at 18°C overnight to produce the native protein. *Ft*TrpAB cultures were treated differently to produce selenomethionine (SeMet)-labeled protein. At an OD_600_ of 1.0, the *Ft*TrpAB cultures were spun down for 30 min at 4000 rev min^−1^. The supernatant was then decanted. LB–PO_4_–glucose pellets (from 4 l culture) were resuspended in 1 l M9 medium (Orion Enterprises, Wheeling, Illinois, USA) supplemented with 0.4%(*w*/*v*) glucose, 13 m*M* NaCl, 0.1 m*M* CaCl_2_, 2 m*M* MgSO_4_, 1%(*w*/*v*) thiamine and antibiotics (Stols *et al.*, 2004 ▸). 0.01%(*w*/*v*) each of l-leucine, l-isoleucine, l-lysine, l-phenylalanine, l-threonine and l-valine were added to inhibit the metabolic pathway of methionine synthesis and encourage SeMet incorporation. The culture was transferred to 18°C, and at an OD_600_ of 1.0 SeMet (90 mg; Orion Enterprises, Wheeling, Illinois, USA) was added. After 15 min, protein expression was induced with 0.5 m*M* IPTG. The cells were incubated at 18°C overnight. The cells were then harvested at 4500 rev min^−1^ for 20 min at 4°C and resuspended in lysis buffer [500 m*M* NaCl, 5%(*w*/*v*) glycerol, 50 m*M* HEPES pH 8.0, 20 m*M* imidazole, 10 m*M* β-mercaptoethanol and protease inhibitor (one tablet per 50 ml of extract; Roche, Mannheim, Germany)] supplemented with 1 m*M* pyridoxal 5′-phosphate (PLP) and stored at −80°C.

SeMet-labeled *Ft*TrpAB and native *Sp*TrpAB were purified using the procedure described previously (Kim *et al.*, 2004 ▸). The harvested cells were thawed and 1 mg ml^−1^ lysozyme was added. This mixture was kept on ice for 20 min with gentle shaking and was then sonicated. The lysate was clarified by centrifugation at 36 000*g* for 1 h and filtered through a 0.45 µm membrane. The clarified lysate was applied onto a 5 ml nickel HisTrap HP column (GE Healthcare Life Sciences) and the His_6_-tagged protein was released with elution buffer (500 m*M* NaCl, 5% glycerol, 50 m*M* HEPES pH 8.0, 250 m*M* imidazole, 10 m*M* β-mercaptoethanol). This was followed by a buffer-exchange step using a customized desalting column (Sephadex G-25 Fine XK 26/20, GE Healthcare Life Sciences) equilibrated with buffer consisting of 20 m*M* Tris–HCl pH 7.5, 500 m*M* NaCl, 2 m*M* DTT. All of these steps were performed using an ÄKTAxpress system (GE Healthcare Life Sciences). The fusion tag was removed by treatment with recombinant His_7_-tagged Tobacco etch virus (TEV) protease. Nickel-affinity chromatography was used to remove the His_6_ tag, uncut protein and His_7_-tagged TEV protease (Blommel & Fox, 2007 ▸). The *Sp*TrpAB ortholog was subjected to an extra purification step via size-exclusion chromatography on a Superdex 200 HiLoad 26/60 column (GE Healthcare Life Sciences) in crystallization buffer (200 m*M* NaCl, 20 m*M* HEPES pH 8.0, 2 m*M* DTT). The *Ft*TrpAB protein was dialyzed against crystallization buffer consisting of 250 m*M* NaCl, 20 m*M* HEPES pH 8.0, 2 m*M* dithiothreitol (DTT) and the proteins were then concentrated to 68 mg ml^−1^ (*Ft*TrpAB) and 33.6 mg ml^−1^ (*Sp*TrpAB) using an Amicon Ultra centrifugal filter device with a 10 000 molecular-weight cutoff (Millipore, Billerica, Massachusetts, USA), flash-cooled in liquid nitrogen and stored at −80°C.

The TrpAB protein concentration was determined spectrophotometrically by measuring the absorbance at 280 nm on a NanoDrop ND-1000 spectrophotometer (Thermo Scientific) against buffer containing an equimolar concentration of PLP. The concentration was calculated using extinction coefficients of 34185 and 39435 *M* ^−1^ cm^−1^, respectively, computed from the amino-acid sequence.

### 2.3. Expression of TrpA and purification for crystallization   

An LB medium starter culture was supplemented with 40 m*M* K_2_HPO_4_ and ampicillin (150 µg ml^−1^) for *LpPh*TrpA and *LpPa*TrpA, grown and shaken overnight at 37°C and 200 rev min^−1^. The starter cultures were used to inoculate 1 l of enriched M9 medium for large-scale SeMet-labeled protein production, which was carried out as described above. From each litre of cell culture, 8 g of cell pellet containing SeMet-labeled *LpPh*TrpA or *LpPa*TrpA protein was obtained and was consequently resuspended in lysis buffer and stored at −80°C.

SeMet-labeled *LpPh*TrpA and *LpPa*TrpA were purified in the same manner as SeMet-labeled *Ft*TrpAB. However, instead of dialyzing these proteins against crystallization buffer, they were buffer-exchanged using an Amicon Ultra centrifugal filter device with a 10 000 molecular-weight cutoff (Millipore, Billerica, Massachetts, USA) with 250 m*M* NaCl, 20 m*M* HEPES pH 8.0, 2 m*M* DTT, flash-cooled in liquid nitrogen and stored at −80°C. Protein concentrations were also determined with a NanoDrop ND-1000 using extinction coefficients of 24870 and 23505 *M* ^−1^ cm^−1^, respectively, computed from the amino-acid sequence.

### 2.4. Expression and purification for enzymatic assays   

For each ortholog, a starter culture was grown overnight at 37°C and 200 rev min^−1^ in LB medium with ampicillin (100 µg ml^−1^) and kanamycin (30 µg ml^−1^) and supplemented with 40 m*M* K_2_HPO_4_. The following morning, 4 l LB–PO_4_–glucose (2 g per litre) medium with antibiotics was inoculated with 30 ml of the overnight culture and was grown at 37°C and 200 rev min^−1^. After reaching an OD_600_ of 1.0 the cultures were transferred to 4°C to cool, and after 1 h the temperature was increased to 18°C. After 15 min, protein expression was induced with 0.5 m*M* IPTG. The cells were incubated at 18°C overnight. The harvested cells containing TrpAB were resuspended in lysis buffer [500 m*M* NaCl, 5%(*w*/*v*) glycerol, 50 m*M* HEPES pH 8.0, 20 m*M* imidazole, 10 m*M* β-mercaptoethanol, protease inhibitor (one tablet per 50 ml of extract), 1 m*M* PLP] and stored at −80°C. All three native proteins were purified using the procedure described above for *Ft*TrpAB. The samples were concentrated to 40 mg ml^−1^ (*LpPh*TrpAB), 40 mg ml^−1^ (*Sp*TrpAB) and 140 mg ml^−1^ (*Ft*TrpAB), flash-cooled in liquid nitrogen in 35 µl droplets and subsequently used in enzymatic assays. *Mt*TrpAB was purified as described previously (Wellington *et al.*, 2017 ▸).

### 2.5. Crystallization   

The *Ft*TrpAB and *Sp*TrpAB proteins were crystallized using sitting-drop vapor diffusion at 16 and 24°C, respectively, in a CrystalQuick 96-well round-bottom plate (Greiner Bio-One North America, Monroe, North Carolina, USA). A 400 nl droplet of the protein (35 or 34 mg ml^−1^) with 1 m*M* PLP and 1 m*M* l-Ser (*Ft*TrpAB) or 0.5 m*M* PLP (*Sp*TrpAB) was mixed with a 200 nl droplet and 400 nl crystallization reagent and allowed to equilibrate against 135 µl crystallization reagent. The nanopipetting was performed using a Mosquito nanolitre liquid-handling system (TTP Labtech, Cambridge, Massachusetts, USA). The plates were then incubated within a RoboIncubator automated plate-storage system (Rigaku). Automated crystal visualization (Minstrel III, Rigaku) was utilized to locate several crystals. The best crystals of SeMet-labeled *Ft*TrpAB were obtained from 0.2 *M* calcium acetate, 0.1 *M* imidazole–HCl pH 8.0, 10%(*w*/*v*) PEG 8000. The *Sp*TrpAB crystals grew from 0.2 *M* ammonium acetate, 0.1 *M* Tris–HCl pH 8.5, 25% PEG 3350.

*LpPh*TrpA (at 25 mg ml^−1^) and *LpPa*TrpA (at 62.5 mg ml^−1^) were screened in the same manner, but without the addition of extra ligands, using a droplet consisting of 400 nl protein solution and 400 nl crystallization reagent that was allowed to equilibrate over 135 µl of the respective reservoir condition. The proteins were screened against the MCSG 1–4 screens (Microlytic) and the Index screen (Hampton Research) at 16°C. The best crystals of SeMet-labeled *LpPh*TrpA were obtained from 0.01 *M* sodium citrate, 33%(*w*/*v*) PEG 6000. The SeMet-labeled *LpPa*TrpA crystals grew from 0.2 *M* sodium chloride, 0.1 *M* bis-Tris pH 6.5, 25%(*w*/*v*) PEG 3350.

### 2.6. Data collection   

The crystals were cryoprotected in their respective mother liquors supplemented with 10% (*Sp*TrpAB, *LpPh*TrpA and *LpPa*TrpA) or 25% (*Ft*TrpAB) glycerol and were subsequently flash-cooled in liquid nitrogen. X-ray diffraction data were collected on the Structural Biology Center 19-ID beamline at the Advanced Photon Source, Argonne National Laboratory. The images were recorded on an ADSC Q315r detector. The data sets were processed with the *HKL*-3000 suite (Minor *et al.*, 2006 ▸). Intensities were converted to structure-factor amplitudes in the *CTRUNCATE* program (French & Wilson, 1978 ▸; Padilla & Yeates, 2003 ▸) from the *CCP*4 package (Winn *et al.*, 2011 ▸). The data-collection and processing statistics are given in Table 1 ▸.

### 2.7. Structure solution and refinement   

The *Sp*TrpAB structure was solved by molecular replacement in *Phaser* (McCoy, 2007 ▸) using the structures of *Sp*TrpA (PDB entry 6qky; unpublished work) and of TrpB from *Bacillus anthracis* (PDB entry 4neg; Center for Structural Genomics of Infectious Diseases, unpublished work). The initial model was autobuilt in *PHENIX* (Adams *et al.*, 2013 ▸) and was further improved by manual correction in *Coot* (Emsley & Cowtan, 2004 ▸) and crystallographic refinement in *PHENIX* (Afonine *et al.*, 2012 ▸). The *Ft*TrpAB, *LpPh*TrpA and *LpPa*TrpA structures were solved by the SAD method using selenium absorption peak data in *SHARP* (Vonrhein *et al.*, 2007 ▸) or *HKL*-3000 (for *Lp*TrpA; Minor *et al.*, 2006 ▸) and were autobuilt in *Buccaneer* (Cowtan, 2006 ▸). The final model was obtained using alternating manual rebuilding in *Coot* and maximum-likelihood refinement in *PHENIX* (Afonine *et al.*, 2012 ▸). The refinement statistics are given in Table 1 ▸.

The atomic coordinates and structure factors have been deposited in the Protein Data Bank under accession codes 5kzm (*Ft*TrpAB), 5kin (*Sp*TrpAB), 5k9x (*LpPh*TrpA) and 5kmy (*LpPa*TrpA).

### 2.8. Preparation of material for kinetic assays   

Prior to kinetic and/or biophysical characterization, *Mt*TrpAB was dialyzed for 2–4 h in TrpAB buffer (20 m*M* HEPES pH 8.0, 100 m*M* KCl, 1 m*M* TCEP, 40 µ*M* PLP) to remove glycerol. After dialysis for 2–4 h, the buffer was exchanged with fresh buffer and dialysis continued overnight. The three other orthologs, however, were stored in 20 m*M* HEPES pH 8.0, 200 m*M* NaCl, 2 m*M* DTT buffer containing no glycerol after purification and did not require dialysis before use.

The compounds F9, F6 and IPP were custom-synthesized by GVK Bio (Cambridge, Massachusetts, USA). The *Mt*TrpAB inhibitor BRD4592 was synthesized internally at the Broad Institute as described previously (Wellington *et al.*, 2017 ▸).

### 2.9. Measurement of enzyme kinetics by UV absorption   

Enzyme kinetics for each ortholog were determined over 30 min under saturating substrate conditions (200 µ*M* indole and 60 m*M* l-Ser) in 1 ml TrpAB buffer. An Agilent Technologies Cary 400 Series UV–Vis spectrophotometer set to 290 nm was used for UV absorption measurements. A baseline reading with no enzyme was established, after which enzyme was added every 2 min to give a final concentration range from 50 n*M* to 2.4 µ*M*. Product progress curves were determined at appropriate enzyme concentrations over a 10 min period in which product generation was linear to determine the *K*_m_ and *k*_cat_ parameters. A value of Δ∊ = 1890 *M* ^−1^ cm^−1^ was used for the indole to l-Trp conversion. In all cases, these enzymes were studied at room temperature (22°C). These experiments were performed on triplicate test occasions with triplicate replicates in each case.

### 2.10. LC-MS assay   

For the liquid chromatography-mass spectrometry (LC-MS) assay, all reagents were prepared in a 96-well plate with a final reaction volume of 50 µl. Compound IC_50_ reactions were run at substrate *K*_m_ conditions (10 µ*M* indole, 20 m*M* l-serine). Compound concentrations ranged from 0 to 200 µ*M*. 10× *K*_m_ substrate solutions were prepared, with 5 µl additions of both indole and serine solutions to the wells. The final concentrations of each protein were as follows: 100 n*M* *Sp*TrpAB, 5 n*M* *Ft*TrpAB, 600 n*M* *LpPh*TrpAB and 100 n*M* *Mt*TrpAB prepared in TrpAB buffer.

Standard curves for l-Trp and indole were included with each mass-spectrometry experiment for quantification purposes only. An l-Ser standard curve was also included as a biological check for each ortholog. Final l-Ser standard curve concentrations included 48, 24, 12, 6, 3, 1.5, 0.75 and 0 m*M* at saturating (500 µ*M*) indole (5× solution at 2.5 m*M* indole with 10 µl additions). After all compound, substrate and standard curve solutions had been prepared, 30 µl of a 1.67× protein solution was added to each well to start the reaction.

After mixing and allowing 10 min incubation at room temperature, the reactions were quenched using 150 µl 0.1% formic acid in methanol followed by storage at 4°C for at least 2 h. The sample plates were then centrifuged for 15 min at 3900 rev min^−1^ (∼3061*g*) and an aliquot of the supernatant was diluted 1:10 with water. 3.75 µl of this final solution was injected and analyzed. l-Trp and indole were detected by UPLC-MS (Waters, Milford, Massachusetts, USA). Compounds were quantified by selected ion recording (SIR) on an SQ mass spectrometer by negative electrospray ionization. The SIR method was set for l-Trp at 203.4 *m*/*z* and for indole at 116.3 *m*/*z*. Mobile phase *A* consisted of 0.1% ammonium hydroxide in water, while mobile phase *B* consisted of 0.1% ammonium hydroxide in acetonitrile. The gradient ran from 2% to 95% mobile phase *B* over 2.65 min at 0.9 ml min^−1^. An Acquity BEH C18, 1.7 µm, 2.1 × 50 mm column was used with the column temperature maintained at 65°C.

### 2.11. Data analysis   

Kinetic experiments were run in triplicate and the reported values represent the average of at least three independent experiments. *K*_m_, *k*_cat_ and IC_50_ data were plotted using *GraphPad Prism* 7.0 and *Origin* 8.0.

## 3. Results   

### 3.1. Protein preparation   

The recombinant tryptophan synthases from the pathogenic bacteria *F. tularensis*, *S. pneumoniae* and *L. pneumophila* Philadelphia have been produced for detailed characterization and comparison with the previously studied enzymes from *S. typhimurium*, *E. coli* and *M. tuberculosis* (Wellington *et al.*, 2017 ▸). The level of pairwise sequence identity between the TrpBs from these organisms ranges from 51% to 59%, with the exception of the *Ft*TrpB/*St*TrpB pair, which show 81% conserved residues. The TrpAs are more variable, with only 25–33% sequence identity for most pairs and 50% for the *Ft*TrpA/*St*TrpA pair (Table 2 ▸), suggesting that there are different evolutionary pressures on the two subunits.

To obtain sufficient amounts of protein-complex samples, TrpA and TrpB were coexpressed from individual vectors in *E. coli*. In all cases, either the TrpA (*Ft*TrpAB and *LpPh*TrpAB) or TrpB (*Sp*TrpAB) subunits were equipped with an N-terminal His_6_ tag, which was subsequently removed by treatment with TEV protease. The resulting proteins carry an additional three N-terminal residues SNA on the tagged subunit. In addition to TrpABs, TrpAs from the *L. pneumophila* strains Paris and Philadelphia (*LpPa*TrpA and *LpPh*TrpA, respectively; 99% identical) have been produced for crystallographic studies, also with a removable N-terminal His_6_ tag. *Ft*TrpAB and *LpPh*TrpA were produced as SeMet-labeled derivatives, while all other proteins were expressed in the native form. The purified proteins were at least 90% pure as judged by PAGE.

### 3.2. Structure determination   

The *Sp*TrpAB protein was crystallized in space group *P*2_1_ with the entire αββα heterotetramer present in the asymmetric unit (Fig. 1 ▸, Table 1 ▸). The structure, which was determined at 2.45 Å resolution, was solved by molecular replacement. In chains *A* and *C*, corresponding to TrpA (amino-acid residues 1–258), residues 1, 180–189 and 182–187, respectively, were not modeled owing to a lack of interpretable electron density. Similarly, in TrpB (amino-acid residues 4–407) the N-terminal SNA sequence and the C-terminal end (residues 403–407) are not present in the respective chains *B* and *D*. The other ortholog, *Ft*TrpAB, crystallized in space group *C*222_1_ and the asymmetric unit contains only one αβ module. This structure was solved by experimental SAD phasing and was refined to 2.80 Å resolution. In *Ft*TrpAB, TrpA (chain *A*; residues 1–269) lacks the N-terminal SNA sequence and residues 183–191, while in TrpB (chain *B*; residues 1–396) the C-terminal residue is not present. For *L. pneumophila* only the TrpA subunit could be crystallized. The *LpPa*TrpA and *LpPh*TrpA structures were determined by experimental SAD phasing at 1.91 and 2.02 Å resolution, respectively. The *LpPh*TrpA protein crystallized in the orthorhombic space group *P*2_1_2_1_2_1_. The asymmetric unit contains one molecule of TrpA and the model lacks the N-terminal SNA residues, residues 57–59, residues 180–186 and the C-terminal residue 272. *LpPa*TrpA also crystallized in space group *P*2_1_2_1_2_1_ with one chain in the asymmetric unit. The N-terminal SN residues and residues 180–187 and 270–273 are missing from the final model.

### 3.3. Kinetic characterization   

**Figure 4:**
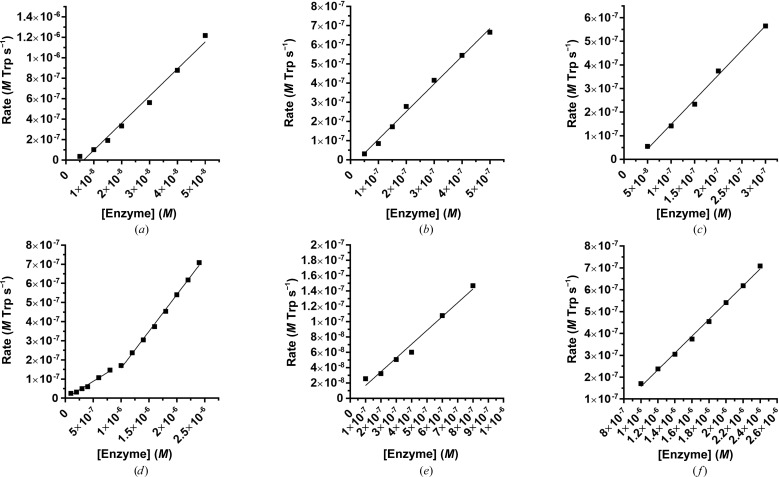
Enzyme versus reaction rate dependency for TrpAB enzymes from (*a*) *F. tularensis* (26 *M* l-Trp s^−1^ *M* ^−1^ enzyme), (*b*) *S. pneumoniae* (1.4 *M* l-­Trp s^−1^ *M* ^−1^ enzyme), (*c*) *M. tuberculosis* (2.0 *M* l-Trp s^−1^ *M* ^−1^ enzyme), (*d*) *L. pneumophila* (all concentrations), (*e*) *L. pneumophila* (low concentrations) (0.17 *M* l-Trp s^−1^ *M* ^−1^ enzyme) and (*f*) *L. pneumophila* (high concentrations) (0.38 *M* l-­Trp s^−1^ *M* ^−1^ enzyme).

Simultaneously with structural characterization, we performed kinetic analyses of the three new orthologs (*Ft*TrpAB, *Sp*TrpAB and *LpPh*TrpAB) and compared them with the *Mt*TrpAB reference. A UV-based assay was used to measure the production of l-Trp from indole and l-Ser. Firstly, the enzyme concentration versus catalytic rate relationship was determined to identify the linear rate dependencies. Both the *Sp*TrpAB and *Ft*TrpAB enzymes displayed specific activities that were comparable to (*Sp*TrpAB, 1.4 *M* l-Trp s^−1^ *M* ^−1^ enzyme) or higher (*Ft*TrpAB, 26 *M* l-Trp s^−1^ *M* ^−1^ enzyme) than that of *Mt*TrpAB (2.0 *M* l-Trp s^−1^ *M* ^−1^ enzyme), with the rate being linearly dependent on enzyme concentration over the entire tested range. The *LpPh*TrpAB enzyme, however, was less active than the *Mt*TrpAB enzyme, displaying a biphasic dependency with both components appearing to be linear. The specific activity at low enzyme concentrations (50–800 n*M*) was much lower (0.17 *M* l-Trp s^−1^ *M* ^−1^ enzyme), while the higher concentration range (1000–2400 n*M*) displayed an improved but still significantly lower specific activity (0.38 *M* l-Trp s^−1^ *M* ^−1^ enzyme) (Fig. 4 ▸). The source of this higher order effect is not obvious, but could be explained by the equilibrium between α subunits and ββ dimers and αββα tetramers, with higher protein concentrations favoring the more active αββα oligomeric state. We have observed such an equilibrium for the *Mt*TrpAB enzyme (Wellington *et al.*, 2017 ▸). The specific activity order is as follows: *Ft*TrpAB >> *Mt*TrpAB, *Sp*TrpAB >> *LpPh*TrpAB.

**Figure 5:**
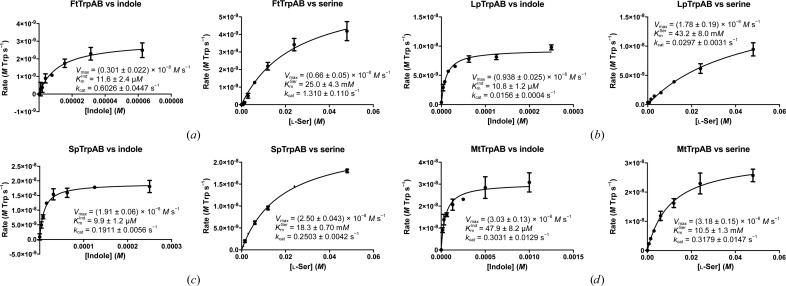
Kinetics of TrpAB orthologs. (*a*) *Ft*TrpAB at 5 n*M*, (*b*) *LpPh*TrpAB at 600 n*M*, (*c*) *Sp*TrpAB at 100 n*M*, (*d*) *Mt*TrpAB at 100 n*M*. The left panels show reaction rates versus indole concentration in the presence of 48 m*M* l-Ser; the right panels show reaction rates versus l-Ser concentration in the presence of 0.5 m*M* indole.

These data were used to set the appropriate enzyme concentrations (5 n*M* *Ft*TrpAB, 100 n*M* *Mt*TrpAB, 100 n*M* *Sp*TrpAB and 600 n*M* *LpPh*TrpAB), resulting in linear l-Trp production progress curves over a 10 min reaction period, to determine the apparent *K*_m_ and *k*_cat_ parameters using the LC-MS assay. The apparent *K*_m_ values are similar across all of the species for both substrates tested (indole and l-Ser). The *k*_cat_ values were reproducible across experiment replicates and substrates, suggesting that saturation was achieved for the independent substrate in each case. The absolute *k*_cat_ values were consistent with the specific activities described above, following the activity order *Ft*TrpAB >> *Mt*TrpAB, *Sp*TrpAB >> *LpPh*TrpAB (Fig. 5 ▸).

### 3.4. Inhibition studies   

**Figure 6:**
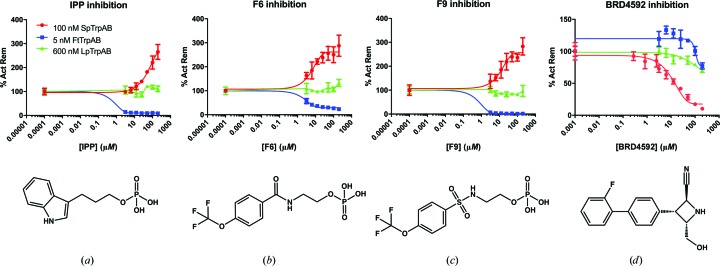
Inhibition of TrpAB orthologs with (*a*) IPP, (*b*) F6, (*c*) F9 and (*d*) BRD4592. Enzyme concentrations for all experiments are shown in (*a*).

In addition, the three TrpAB orthologs were profiled against the reported commercially available inhibitors F9 [*N*-(4′-trifluoromethoxybenzenesulfonyl)-2-aminoethyl phosphate; CID identifier 16122526], F6 [*N*-(4′-trifluoromethoxy­benzoyl)-2-aminoethyl phosphate; CID identifier 16122525] and IPP (indole­propanol phosphate; CID identifier 3713), as well as the recently discovered *Mt*TrpAB inhibitor BRD4592 (CID identifier 54650477; Wellington *et al.*, 2017 ▸) (Fig. 6 ▸). The LC-MS-based assays examined inhibition of the β reaction with indole and l-Ser as substrates. F9 was found to be a potent inhibitor (IC^β^_50_ = 114 n*M*) of *Ft*TrpAB under substrate *K*_m_ conditions (10 µ*M* indole, 20 m*M* l-Ser), while only slightly inhibiting *LpPh*TrpAB. Interestingly, F9 appears to be an activator of *Sp*TrpAB (Fig. 6 ▸). A similar profile is again exhibited with F6 and IPP, whereby inhibition was only observed for the *Ft*TrpAB enzyme, with IC^β^_50_ = 1.46 µ*M* for F6 and IC^β^_50_ = 0.08 µ*M* for IPP. A different profile was seen when using the *Mt*TrpAB inhibitor BRD4592. All three orthologs are slightly inhibited; however, a measureable IC_50_ was only obtained for the *Sp*TrpAB ortholog (IC^β^_50_ = 21 µ*M*) (Fig. 6 ▸).

## 4. Discussion   

### 4.1. Structural comparison with other TrpAB orthologs   

**Figure 7:**
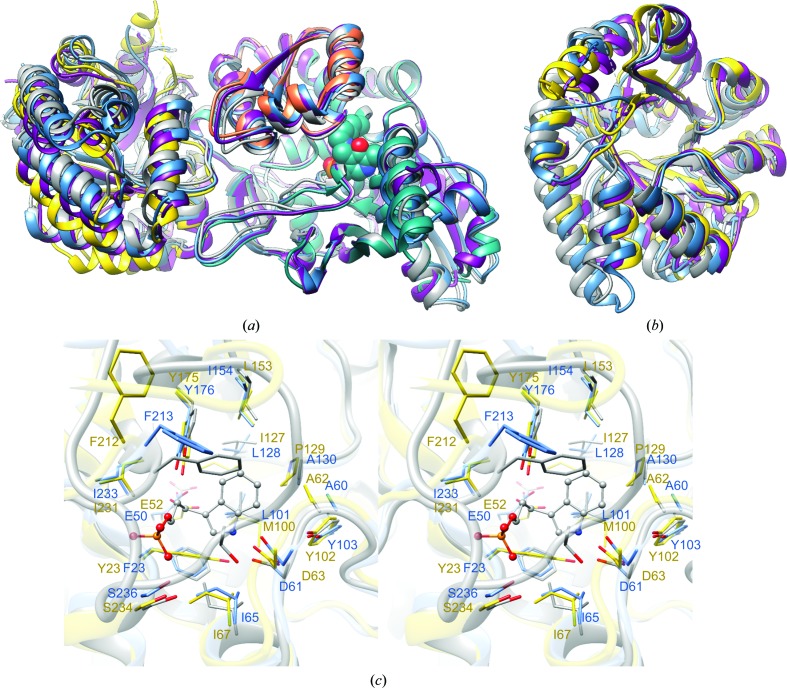
Comparison of TrpAB orthologs. (*a*) Superposition of *Sp*TrpB (yellow, TrpA, chain *C*; coral/cyan, TrpB, chain *D*) with *Ft*TrpB (blue), *Mt*TrpB (purple; chains *A* and *B*; PDB entry 5tcf; Wellington *et al.*, 2017 ▸) and *St*TrpB (gray; PDB entry 1bks; Rhee *et al.*, 1996 ▸). PLP from *Sp*TrpAB is shown in a sphere representation. TrpA is shown to indicate the mutual orientation of the subunits. (*b*) Superposition of TrpA extracted from the TrpAB heterodimers. (*c*) Stereoview of the TrpA active-site superposition of *Sp*TrpA (yellow), *Ft*TrpA (blue) and *St*TrpA in complex with IPP (gray; PDB entry 1qop; Weyand & Schlichting, 1999 ▸).

We have determined the structures of the *Ft*TrpAB and *Sp*TrpAB αββα heterotetramers and of the α subunits *LpPa*TrpA and *LpPh*TrpA. The overall structures of the complexes, along with the α subunits, are essentially identical to those of the orthologs characterized previously, with the heterotetramer representing the complete functional unit (Fig. 1 ▸). Despite the rather low sequence identity of the TrpAs, the three polypeptides superpose with r.m.s.d.s of 1.4–1.9 Å amongst themselves and with the orthologs *Mt*TrpA or *St*TrpA (Table 2 ▸, Fig. 7 ▸). The enzyme from *F. tularensis*, which is the most closely related to *St*TrpAB, shows even better agreement, with an r.m.s.d. of 0.8 Å for corresponding *St*TrpA C^α^ atoms. A similar pattern is observed for the TrpBs, which overlap with r.m.s.d.s of 0.7–1.0 Å.

As expected in the absence of any TrpA ligand, the α subunit adopts an open conformation with a disordered loop αL6, regardless of whether the subunit is complexed with TrpB or alone. In isolated *LpPh*TrpA parts of loop αL2 could not be modeled, indicating its high flexibility. The TrpA binding pocket and these critical loops are generally well conserved in terms of composition, including the catalytic residues, one of which is provided by loop αL2. One important feature, although only noted at the sequence level owing to disorder, is the lack of conservation in the N-terminal region of loop αL6. In the *Salmonella* enzyme this section carries αArg179, which has been shown to provide loop stabilization via hydrogen bonds between the guanidinium group and the main-chain atoms (Schneider *et al.*, 1998 ▸). With the exception of *Ft*TrpA, this residue is replaced by much smaller and in some cases hydrophobic residues, Ile in *Sp*TrpA, Leu in *Lp*TrpA and Thr in *Mt*TrpA, and cannot form interactions equivalent to those of αArg179. It has previously been shown that an αArg179Leu mutation reduces the affinity of the substrate IGP for *St*TrpA and slows the TrpAB reaction (Brzović *et al.*, 1993 ▸). It is not clear that this is a valid assumption for the other orthologs; however, *Mt*TrpAB indeed has a higher *K*_m_ for IGP than *St*TrpA. In addition, it is also consistent with the relative rank order of specific activities observed across this panel of TrpAB orthologs, although only in the context of the β reaction.

Within the ordered fragments of the TrpA pocket, some sequence variability is observed at the positions of αPro129*Sp* (the equivalent residues are αPro135*Mt*, αAla130*Ft*, αAla129*St* and αVal129*Lp*), αMet100*Sp* (αMet100*Lp* and αMet106*Mt* but αLeu101*Ft* and αLeu100*St*) and αTyr23*Sp* (replaced by Phe in *Ft*TrpA, *Lp*TrpA and *St*TrpA). Notably, though, despite the good superposition of the main-chain atoms throughout most of the subunit, the side chains adopt slightly different conformations (Fig. 7 ▸). The most pronounced discrepancy is observed for αPhe212*Sp*, a residue that T-stacks against the aromatic ring of indole in the ligand-bound *St*TrpA state (Weyand & Schlichting, 1999 ▸). The position of this residue is affected by the mobile αL6 loop in the substrate-bound closed state that reinforces the proper placement of the Phe side chain with respect to the substrate moiety. Without such constraints, in *Sp*TrpA, as well as in *Lp*TrpA, it points somewhat outside of the binding pocket towards the helical layer of the protein. In *Ft*TrpA it is oriented more towards the cavity, but its position is still halfway from the state achieved in the substrate-bound complex (Fig. 7 ▸). Interestingly, this residue is replaced by αLeu218 in the *Mt*TrpA ortholog, where it also swings outside the binding pocket. The catalytic αGlu52*Sp* and its equivalents in other orthologs also display some conformational diversity; in some cases, such as *Ft*TrpA or *St*TrpA, it points towards the protein core, while in others (*Sp*TrpA and *Mt*TrpA) it faces the binding pocket. There are no apparent structural differences between TrpA in the TrpAB complex versus TrpA alone. The only exception is a slight movement of loop αL2 towards the active site of TrpA in the αβ heterodimer unit.

In our *Ft*TrpAB and *St*TrpAB structures the β subunits exist in the open conformation, or more precisely in the expanded open conformation β^eO^ reported previously for several *St*TrpAB structures [PDB entries 2j9z (Blumenstein *et al.*, 2007 ▸), 1qoq (Weyand & Schlichting, 1999 ▸) and 1kfb (Kulik *et al.*, 2002 ▸)], the *P. furiosus* ortholog [PDB entries 5e0k (Buller *et al.*, 2015 ▸) and 1wdw (Lee *et al.*, 2005 ▸)] and *Mt*TrpAB (PDB entry 5tcf; Wellington *et al.*, 2017 ▸), suggesting that this state may be more common than previously indicated. The active site carries a PLP moiety covalently attached to βLys91*Sp* (βLys86*Ft*, βLys101*Mt*). The β active site is very conserved both in terms of sequence and the conformation of the PLP cofactor and side chains, with a few exceptions. *Ft*TrpB and *Sp*TrpB share an Ala with *St*TrpB (βAla84, βAla89 and βAla85, respectively), but *Mt*TrpB has an equivalent βSer99 that makes a direct hydrogen bond to PLP. This interaction is missing in the other three orthologs. βThr87 is present in *Sp*TrpB (and βThr97 in *Mt*TrpB), which is replaced by glycine in *Ft*TrpB and *St*TrpB. There is no obvious role for this substitution. Two important catalytic residues, threonine (βThr114*Sp*, βThr109*Ft*, βThr124*Mt* and βThr110*St*) and aspartic acid (βAsp310*Sp*, βAsp304*Ft*, βAsp319*Mt* and βAsp305*St*), show a very different conformational behavior in the open state of β-subunit orthologs. The threonine, which is involved in coordination of the substrate/product carboxylate, shows nearly the same conformation in all four orthologs, while the conformations of the aspartic acid, which is involved in interaction with the amino group of the reagents, are very different. Larger conformational diversity is also observed for βGln118, a residue that is conserved in all four enzymes. However, only in *Mt*TrpB does this residue form a direct hydrogen bond to O3 of the PLP cofactor. The side chains of a few other residues (βGln89, βSer234 and βLys381 in *Ft*TrpB) also show somewhat different conformations, but these are much less pronounced. The phosphate group of PLP is anchored by interaction with the N-terminal dipole of helix βH9, direct hydrogen bonds to several main-chain amino groups (helix βH9 and a short loop between βS7 and βH9) and three conserved side chains (βHis85, βSer234 and βAsn235 in *Ft*TrpB and βHis90, βSer240 and βAsn241 in *Sp*TrpB). These small changes in sequence and conformational propensity may explain the differences in substrate affinities and reaction rates.

**Figure 8:**
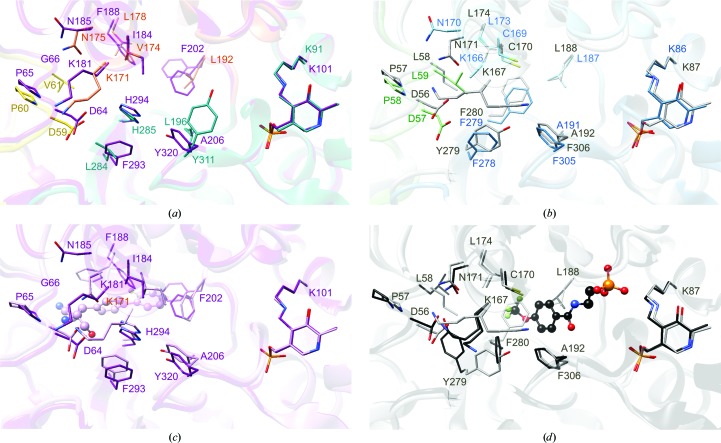
Comparison of the intersubunit tunnel. (*a*) Superposition of *Sp*TrpAB (yellow, TrpA, chain *C*; coral/cyan, TrpB, chain *D*) with ligand-free *Mt*TrpAB (purple; chains *A* and *B*; PDB entry 5tcf; Wellington *et al.*, 2017 ▸). (*b*) Superposition of ligand-free *Mt*TrpAB (purple; chains *A* and *B*; PDB entry 5tcf) with *Mt*TrpAB in complex with BRD4592 (pink; chains *C* and *D*; PDB entry 5tci; Wellington *et al.*, 2017 ▸). Note that in the shown chain *D* TrpB adopts the β^O^ state. Chain *B* exists as a mixture of the β^O^ and β^eO^ states. (*c*) Superposition of *Ft*TrpAB (green, TrpA; blue/navy, TrpB) with *St*TrpAB (gray; PDB entry 1bks; Rhee *et al.*, 1996 ▸). (*d*) *St*TrpAB (gray; PDB entry 1bks) with *St*TrpAB in complex with F6 (black; PDB entry 4wx2; Hilario *et al.*, 2016 ▸). Key residues are shown in stick representation; inhibitors are shown in ball-and-stick representation.

The structures of the *Ft*TrpAB and *Sp*TrpAB αββα heterotetramers provide a new set of high-quality models and enable comparison of the intermolecular tunnel connecting the TrpA and TrpB catalytic pockets. In contrast to the active sites, the composition of the tunnel, which is mostly encompassed by TrpB, varies between the orthologs (Fig. 8 ▸), although generally *Sp*TrpAB shares some features with *Mt*TrpAB while *Ft*TrpAB is similar to *St*TrpAB. This is consistent with the relative specific activities and the conservation of local primary sequence. The cross-comparisons indicate a number of differences. For example, one side of the *Sp*TrpB tunnel contains βTyr311, βHis285 and the neighboring βLeu284, with the tyrosine rotated towards the active site of TrpB, where it could potentially interfere with the β reaction. The opposite side contributes βVal174, βLeu178 and βLeu192. In *Ft*TrpB all of the former residues are replaced by phenyl­alanines (βPhe305, βPhe279 and βPhe278, respectively), while the leucines are conserved and βVal174*Sp* is replaced by βCys169*Ft*. A similar scenario is present in *St*TrpB (βPhe306 and βPhe280), with the exception of βTyr279*St*, which substitutes for βPhe278*Ft*. In *Mt*TrpB the equivalent residues are βTyr320, βHis294 and βPhe293, resembling the *Sp*TrpB composition, but in this case the tyrosine ring points in a different direction, making a hydrogen bond to βHis294. Such an arrangement would be more constrained in *Sp*TrpB owing to the proximity of βLeu196, a residue that is substituted by a much smaller Ala in the other enzymes. *Mt*TrpB also contains phenylalanines (βPhe188 and βPhe202) instead of the leucines that are conserved in the three other TrpBs, and βIle184*Mt* takes the place of βVal174*Sp*. Previous data for the *St*TrpB ortholog showed that large side chains, such as Phe or Trp, in this position hamper indole channeling (Anderson *et al.*, 1995 ▸; Schlichting *et al.*, 1994 ▸; Weyand & Schlichting, 2000 ▸). Therefore, it appears that these variations in the residues composing the tunnel may have a direct impact on the rate of indole transfer and influence the kinetic activities of these enzymes. This may represent a fine-tuning of the enzyme activity without directly involving the residues in the catalytic sites.

Generally, the tunnel displays some level of flexibility and can adapt to enable indole translocation or to specifically bind certain inhibitors. For instance, we showed previously that in *Mt*TrpAB βPhe188 changes conformation to accommodate BRD4592 (Wellington *et al.*, 2017 ▸) both in the open and closed states of the β subunit, while in *St*TrpAB βPhe280 and βTyr279 swing away to provide space for the F6 molecule (Hilario *et al.*, 2016 ▸) in the open state (Fig. 8 ▸). The latter work also proposed that the indole moiety enters TrpB in the vicinity of αLeu21*St* (conserved as αLeu24*Sp*, αLeu34*Mt* and αLeu20*Ft*), βLeu174*St* and βPhe280*St*, which need to move to open up a farther segment of the channel that is lined with residues that do not present major obvious obstacles. In principle, an analogous mechanism can be envisioned for the very similar enzyme from *F. tularensis*. In the other two orthologs alternative mechanisms are most likely to exist. In the *Sp*TrpB/*Mt*TrpB structures, in which βPhe280*St* is replaced by a histidine, this residue adopts a conformation that is compatible with an open channel both in the β^O^ (*Sp*TrpB/*Mt*TrpB) and β^C^ (*Mt*TrpB) states. Moreover, in *Mt*TrpB such an architecture is stabilized by a hydrogen bond to βTyr320*Mt* (in β^O^ and β^C^) and another to βAsn185*Mt* (in *Mt*β^C^), suggesting that it represents the most common conformational state. An analogous interaction with asparagine might be created in *Sp*TrpB upon subunit closure, while His–Tyr bonding would require the concomitant movement of βTyr311*Sp* and βLeu196*Sp*. This coordinated movement is potentially a necessary step for the COMM-domain shift and TrpB closure, as otherwise βLeu170*Sp* would clash with βTyr311*Sp*. On the other hand, the mycobacterial enzyme may need to undergo a different adjustment on the opposite side of the tunnel. Here, there are two bulkier phenylalanine residues, βPhe188 and βPhe202. In both cases these residues appear to be mobile, as in some structures of *Mt*TrpAB βPhe202 exists in double conformations while βPhe188 has been shown to rotate in the complex with the BRD4592 inhibitor. However, for βPhe188 in this alternative state the access from subunit α is blocked; thus, it is possible that the ligand-free conformation of βPhe188 corresponds to the open-tunnel state with only a minor adjustment required.

### 4.2. Allosteric contacts   

Previous investigations of allosteric communication between the TrpAB subunits recognized a number of key interactions at the α–β interface that transmit activation signals. One of them is the main-chain–main-chain hydrogen bond between βSer178 and αGly181 in *St*TrpAB (Spyrakis *et al.*, 2006 ▸; Schneider *et al.*, 1998 ▸). The former residue is preserved in *Ft*TrpB; however, the other two orthologs contain valine. On the other hand, the glycine residue (αGly181*Sp*, αGly182*Ft* and αGly187*Mt*) belongs to the highly conserved GVTG motif of the αL6 loop. In the *S. typhimurium* TrpA α^C^ state the conserved threonine residue from this motif, αThr183, binds through its hydroxyl group to the carboxylate of the catalytic αAsp60 (αAsp61*Sp*, αAsp63*Ft* and αAsp68*Mt*), in addition to the main-chain–main-chain interaction with the αL2 loop. Deletions or point mutations within the αL6 loop, such as αThr183Ala in *St*TrpA, dramatically reduce the α-subunit activity (Yang & Miles, 1992 ▸). Similar modifications in the αL2 loop, including changes to αPro57*St* (αPro60*Sp*, αPro58*Ft* and αPro65*Mt*) and αAsp56*St* (αAsp59*Sp*, αAsp57*Ft* and αAsp64*Mt*) reduce TrpA activity, although significant effects only occur in the context of the TrpAB complex, *i.e.* not when the α subunit alone is assayed (Ogasahara *et al.*, 1992 ▸; Rowlett *et al.*, 1998 ▸).

In the available β^O^ and β^C^ states of the mycobacterial enzyme, the side chain of αAsp64*Mt* (the main chain of αSer63 in β^eO^) interacts with βLys181 from the COMM domain, while the carbonyl group of αAsp68 binds to βArg189 in some of the subunits, as seen before in the *St*TrpA ortholog (Weyand & Schlichting, 1999 ▸). In the *Sp*TrpAB β^eO^ state there is also a hydrogen bond between the αSer58 carbonyl group and βLys171, but βArg179 is too distant to interact with the catalytic aspartate. None of these contacts is observed in the reported *Ft*TrpAB structure, either owing to disorder or to longer distances between the relevant atoms.

Overall, the available data suggest that the geometry and contacts established by loops αL6 and αL2 have a pronounced effect on the enzyme activity. Transition from β^O^ to β^C^ triggers the closure of αL6, which, together with the αL2 and βH6 elements, activates the catalytic aspartate residue. Changes in these elements or in their neighborhood possibly lock αL6 into a low-activity open state (Spyrakis *et al.*, 2006 ▸), thus preventing the proper positioning of the catalytic aspartic acid. Simultaneously with the α-subunit malfunction, destabilization of the αL2–βH6 interactions in mutants reduces the β-subunit activity (Ogasahara *et al.*, 1992 ▸), with the detrimental effect partly alleviated by cation binding. Monovalent cations have been shown to stabilize the *St*TrpAB enzyme, with large cations (Cs^+^ and NH_4_^+^) exhibiting the most pronounced effect (Rowlett *et al.*, 1998 ▸). These effects might result from the chain of interactions linking αL2 to βH6 and further, via the monovalent cation-binding site (MVC), to the active site of the β subunit. The MVC is established by a set of residues localized in the proximity of the channel and the active site of TrpB, which interact with the cation through four main-chain carbonyl moieties (in *S. typhimurium* and *M. tuberculosis*) and a threonine side chain (only in *M. tuberculosis* owing to the presence of Pro in the equivalent position in *St*TrpB). While no monovalent cations have been modeled in the current structures, by analogy to the data collected from the *Mt*TrpAB and *St*TrpAB systems the MVC must be created by βTyr311*Sp*, βGly313*Sp*, βAla273*Sp*, βGly237*Sp* and βThr275*Sp* in *Sp*TrpAB and βPhe305*Ft*, βSer307*Ft*, βGly267*Ft*, βGly231*Ft* in *Ft*TrpAB, with βPro269*Ft* replacing the threonine residue. Depending on the size of the cation, either all residues equivalent to those in *St*TrpAB and *Mt*TrpAB would be involved in cation binding, or only a subset, where the unfilled valencies in the coordination sphere may be completed by water molecules. As mentioned above, the MVC is indirectly connected to the βH6 element of the COMM domain and to TrpA via either a histidine (βHis285*Sp* and βHis294*Mt*) or a phenylalanine (βPhe279*Ft*, βPhe280*St*), switching between hydrophobic Phe–Phe contacts (*Ft*TrpB and *St*TrpB) and the well defined His–Tyr hydrogen bond seen in *Mt*TrpB and most likely to be present in the activated form of *Sp*TrpB. It is not clear how this different organization of the MVC and its interactions with other structural elements affect the sensitivity of the protein to different cations or how the signal transduction is affected.

### 4.3. Enzymatic properties   

In the β-elimination reaction of TrpAB, with a *k* ^β^_cat_ of between 1.7 and 78.6 min^−1^, all of the investigated enzymes appear to be poorer catalysts of the indole-to-tryptophan conversion than the previously studied *Mt*TrpAB (*k* ^β^_cat_ = 197 min^−1^; Wellington *et al.*, 2017 ▸ 1), *Ec*TrpAB (348 min^−1^; Lane & Kirschner, 1983 ▸) and *St*TrpAB (288 min^−1^; Raboni *et al.*, 2007 ▸), at least under the given experimental conditions: at room temperature (20–22°C) at pH 7.6–8.0 in the presence of potassium ion. Similarly, the *K*_m_ for serine is at least 35 times higher for the *Sp*TrpAB and *Ft*TrpAB enzymes (18.3–43.2 m*M*) than for those previously characterized (0.37, 4.4 and 0.58 m*M* for *Ec*TrpAB, *Mt*TrpAB and *St*TrpAB, respectively). Interestingly, however, the *K*_m_ for indole is at least approximately three times lower for all of the currently tested orthologs than those reported for *Mt*TrpAB and *St*TrpAB and is comparable to that of *Ec*TrpAB.

### 4.4. Inhibition   

Several inhibitors have been designed to study the mechanistic details of TrpAB. A number of them are competitive indole-3-glycerol phosphate analogs that bind to subunit α, such as IPP and similar indole-3-alkyl 1-phosphates (Kirschner *et al.*, 1975 ▸), indole-3-acetyl amino acids (Marabotti *et al.*, 2000 ▸) or aryl compounds linked via an amide/sulfonamide/thioether/thiourea to a phosphoalkyl moiety (Ngo, Harris *et al.*, 2007 ▸; Sachpatzidis *et al.*, 1999 ▸). The IC_50_ parameters for these inhibitors against TrpAB have not been determined, with the exception of thioether-linked substrate analogs (Sachpatzidis *et al.*, 1999 ▸), which showed nanomolar values for the α reaction of *St*TrpAB. In addition to competitive inhibition of the α reaction, some of the α-binders, for example indole-3-acetyl-amino acids, IPP and F9, exert allosteric effects on subunit β (Marabotti *et al.*, 2000 ▸; Ngo, Harris *et al.*, 2007 ▸). The more promiscuous ligand F6 has been found to bind not only to the active site of TrpA but also to the intersubunit tunnel, close to the β active site (Hilario *et al.*, 2016 ▸). The influence of competitive inhibitors of TrpA on the TrpB reaction has been linked to their ability to remodel the α site, with the higher degree of ordered TrpA structure triggering more pronounced changes in TrpB (Ngo, Harris *et al.*, 2007 ▸).

Here, we have tested the commercially available compounds IPP, F6 and F9 against the β reaction. Notably, we observed potent inhibition only for *Ft*TrpAB, which is the most similar to the prototypical *St*TrpAB of all the tested enzymes. It therefore seems that the allosteric effect influencing the activity of TrpB is sensitive to local sequence variations and structural features, and consequently might be unique to a subset of orthologs. Alternatively, it is also possible that the lack of TrpB susceptibility originates directly from the poor affinity of these inhibitors for TrpA, but we have not investigated such a scenario biochemically. From a structural perspective, the TrpA active sites are similar enough to at least bind to the very close substrate mimetic IPP, suggesting that the former argument for the lack of inhibition is more likely. Another explanation of these differences involves long-distance effects within and between subunits. The activation of *Sp*TrpAB by the α-binders is unexpected and surprising. However, allosteric sites serve modulatory purposes and a single binding pocket may exert activatory or inhibitory roles. It is therefore possible that the binding of the same ligands to various TrpAB orthologs may result in opposite kinetic effects because of small sequence variations.

In agreement with our previous work demonstrating that BRD4592 inhibition is limited to orthologs containing a glycine residue in the αL2 loop of TrpAB, such as in the case of the *Mt*TrpAB enzyme, no significant effect was observed for all of the tested synthases. The weak inhibition of *Sp*TrpAB, which carries the smallest side chain among the tested enzymes (αVal61 in place of αGly66 in *Mt*TrpAB, αLeu59 in *Ft*TrpAB and αMet58 in *LpPh*TrpAB), supports the previous conclusion that any substitution in the loop would drastically reduce the size of the BRD4592 binding pocket, limiting the inhibitor affinity.

## 5. Conclusions   

Tryptophan synthases have been shown to be conditionally essential enzymes in a number of important human pathogens, but the enzymes of the family have remained unexplored beyond a limited number of representatives. To broaden our perspective on TrpABs, we have purified and characterized three enzymes from *L. pneumophila*, *F. tularensis* and *S. pneumoniae* to uncover the potential unique features of TrpABs and to support future drug-discovery efforts. X-ray crystallography and biochemical studies show a remarkable structural conservation of the architecture and the catalytic and allosteric sites of the enzyme, suggesting preservation of the catalytic mechanism and regulation. At the same time, these enzymes display local sequence and structural differences in the catalytic, allosteric and metal-binding sites. These enzymes also exhibit differences in kinetic properties and their response to inhibitors, yet they display some correlations between biochemical properties and sequence/structural conservation. Notably, not all enzymes were inhibited by the tested compounds. In fact, for the *S. pneumoniae* ortholog the reaction was more efficient in the presence of α-binders. Some of the differences can be explained structurally; however, others may result from the altered conditions in which these enzymes operate *in cellulo*. Nevertheless, understanding these dissimilarities may provide a basis for the design of new species-specific tryptophan synthase inhibitors against both the α and β active sites as well as the allosteric sites, which show higher conformational and sequence variability. Recognition that the targeting of unique allosteric sites may have species-specific effects may be important for the treatment of coexisting infections.

## Supplementary Material

PDB reference: *Ft*TrpAB, 5kzm

PDB reference: *Sp*TrpAB, 5kin

PDB reference: *LpPh*TrpA, 5k9x

PDB reference: *LpPa*TrpA, 5kmy

## Figures and Tables

**Figure 3:**
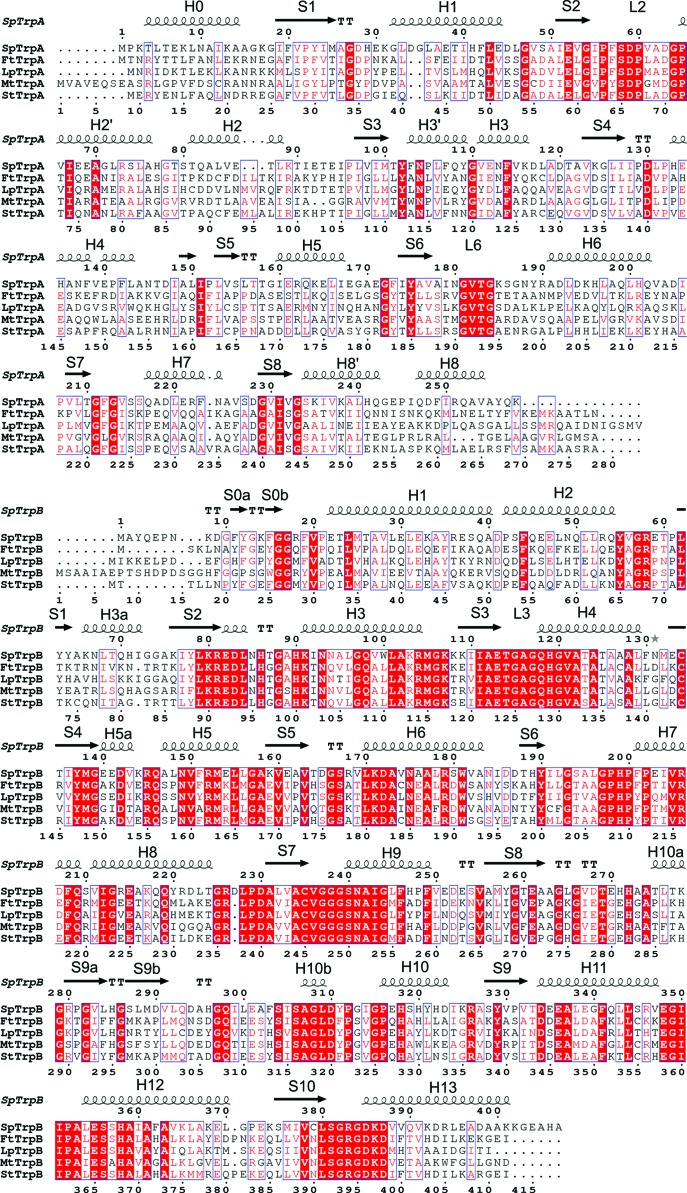
Sequence alignment of TrpA (top) and TrpB (bottom). Sequences are shown for *S. pneumoniae*, *F. tularensis*, *L. pneumophila* Philadelphia, *M. tuberculosis* and *S. typhimurium*. The depicted secondary-structure elements are derived from the *Sp*TrpAB structure.

**Table 1:** Data-processing and refinement statistics Values in parentheses are for the highest resolution shell.

Structure	*Sp*TrpAB	*Ft*TrpAB	*LpPh*TrpA	*LpPa*TrpA
Data processing				
Wavelength (Å)	0.9793	0.9793	0.9792	0.9786
Resolution range (Å)	50.00–2.45 (2.49–2.45)	30.00–2.80 (2.85–2.80)	50.00–2.00 (2.03–2.00)	40.00–1.91 (1.93–1.91)
Space group	*P*2_1_	*C*222_1_	*P*2_1_2_1_2_1_	*P*2_1_2_1_2_1_
Unit-cell parameters				
*a* (Å)	67.70	111.18	47.02	43.71
*b* (Å)	71.16	171.99	71.05	69.65
*c* (Å)	138.68	76.11	71.82	75.08
β (°)	101.69			
Unique reflections	48447	18264	16344	18317
Merged reflections	2386	902	751	858
Multiplicity	3.6 (3.3)	4.8 (4.9)	11.3 (8.4)	4.2 (3.1)
Completeness (%)	99.7 (99.8)	100.0 (100.0)	99.4 (93.1)	99.7 (96.9)
Mean *I*/σ(*I*)	14.3 (1.6)	5.8 (1.5)	38.0 (1.9)	20.0 (2.0)
Wilson *B* factor (Å^2^)	38.97	57.25	24.07	17.55
*R*_merge_ †	0.141 (0.740)	0.132 (0.981)	0.058 (0.893)	0.062 (0.490)
CC_1/2_ ‡	0.683	0.566	0.793	0.703
Refinement				
Resolution range (Å)	49.13–2.45	29.50–2.80	39.21–2.02	37.54–1.91
Reflections (work/test)	45342/2264	32767/1689	13868/1411	30107/1521
*R*_work_/*R*_free_ §	0.181/0.228	0.183/0.235	0.191/0.238	0.176/0.207
No. of non-H atoms				
Total	10029	4987	2184	2245
Macromolecules	9902	4971	2054	2041
Ligands	18	6	0	0
Solvent	109	10	130	204
No. of protein residues	1297	655	262	262
R.m.s.d., bonds (Å)	0.002	0.003	0.003	0.005
R.m.s.d., angles (°)	0.51	0.57	0.66	0.90
Ramachandran statistics¶				
Favored (%)	97.03	94.89	98.47	98.45
Allowed (%)	2.81	4.95	1.53	1.55
Outliers (%)	0.16	0.15	0.0	0.0
Rotamer outliers (%)	1.28	1.17	0.0	3.64
Clashscore	2.53	5.12	1.70	4.40
Average *B* factor (Å^2^)				
Overall	49.14	56.09	34.33	22.30
Macromolecules	49.21	56.10	33.83	21.30
Ligands	63.01	75.38		
Solvent	40.13	38.11	41.43	32.32
No. of TLS groups	20	9	—	6
PDB entry	5kin	5kzm	5k9x	5kmy

† *R*_merge_ = 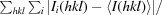 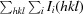, where *I_i_*(*hkl*) is the intensity of observation *i* of reflection *hkl*.

‡ As defined by Karplus & Diederichs (2012 ▸).

§ *R* = 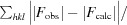 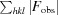 for all reflections, where *F*_obs_ and *F*_calc_ are the observed and calculated structure factors, respectively. *R*_free_ is calculated analogously for the test reflections, which wre randomly selected and excluded from the refinement.

¶ As defined by *MolProbity* (Chen *et al.*, 2010 ▸).

**Table 2:** Primary structure identity and structural similarity between orthologous TrpA and TrpB The first number corresponds to the percentage sequence identity (calculated in *EMBOSS Needle*; Rice *et al.*, 2000 ▸), followed by r.m.s.d. (in Å) for C^α^-atom superposition for the number of pairs given in parentheses (calculated in *CCP*4; Winn *et al.*, 2011 ▸, Krissinel & Henrick, 2004 ▸).

	*Sp*TrpB	*Ft*TrpB	*LpPh*TrpB	*Mt*TrpB	*St*TrpB
*Sp*TrpA		53, 0.87 (385)	57	54, 0.85 (389)	53, 1.02 (377)
*Ft*TrpA	29, 1.89 (233)		53	51, 0.82 (381)	81, 0.65 (388)
*LpPh*TrpA	32, 1.71 (230)	32, 1.38 (244)		59	53
*Mt*TrpA	31, 1.81 (241)	25, 1.37 (244)	33, 1.64 (240)		51, 1.01 (381)
*St*TrpA	29, 1.74 (225)	58, 0.84 (253)	31, 1.36 (240)	26, 1.56 (244)	

## Footnotes

1 Note that in the current experiments the parameters for *Mt*TrpAB are somewhat worse than in the previous study, either owing to slow deterioration of the protein at −80°C or variations in the experimental setup.
